# Plateau Iris – Therapeutic options and functional results after treatment

**DOI:** 10.22336/rjo.2017.22

**Published:** 2017

**Authors:** Crenguța Feraru, Andrei Bâlha, Victor Aursulesei, Andrei Filip, Anca Pantalon

**Affiliations:** * “Gr. T. Popa” University of Medicine and Pharmacy, Iași, Romania; ** Department of Ophthalmology, “Sf. Spiridon” University Hospital, Iași, Romania

**Keywords:** plateau iris, therapy options

## Abstract

We present the therapeutic options and functional results in patients with plateau iris (syndrome or configuration) in consecutive case series.

**Material and method:** Our study included newly diagnosed patients with acute angle closure by “plateau iris” (configuration or syndrome), between June 2016 and April 2017. Series of 8 consecutive patients met the inclusion criteria, all being females. All the patients underwent an individualized treatment according to the underlying mechanism and evolution. Functional results (visual acuity, IOP, topical medication) were reported in the current paper.

**Results:** For 10 months, we diagnosed 14 eyes, from 9 patients with acute angle closure by Plateau Iris, distributed as it follows: 6 eyes with closed angle glaucoma (optic disk and visual field changes), 8 eyes with plateau iris syndrome and 2 eyes with plateau iris configuration. 7/ 8 patients were misdiagnosed with primary open angle glaucoma, whereas only one patient had the correct diagnosis of closed angle glaucoma and underwent peripheral laser iridotomy. As treatment options in our study, we recommended and performed argon laser peripheral iridoplasty + iridotomy in 10/ 14 eyes, cataract lens was extracted in 4 eyes and then replaced with PC-IOL, whereas 2 eyes required a filtering anti-glaucoma surgery (trabeculectomy + PI). 2 eyes from the same patient could not be treated as intended as the patient refused the treatment. In this unique case, Pilocarpine (4%) was temporarily indicated.

**Conclusion:** Plateau iris represents a diagnostic trap, but based on a thorough gonioscopic examination and a good patient history, the right diagnosis can be made, all along with a correct therapeutic approach.

## Introduction

Plateau Iris syndrome is a relatively uncommon form of primary angle closure glaucoma that is seen more often than pupillary block angle in younger adults (3rd or 4th decade) [**1**]. Plateau Iris syndrome is defined as a persistently narrow angle capable of closure in spite of a patent iridotomy. Plateau Iris is an ocular condition that requires appropriate diagnosis and treatment in order to prevent vision loss. The early recognition and intervention are key components to a good overall prognosis in this patient population [**2**]. Patients may develop angle closure, either spontaneously or after pupillary dilation [**3**], but more commonly, patients are asymptomatic and the diagnosis is made on a thorough gonioscopic examination. Additionally, AS-OCT and UBM enhance the diagnosis in plateau iris syndrome/ configuration. 

**Fig. 1:**
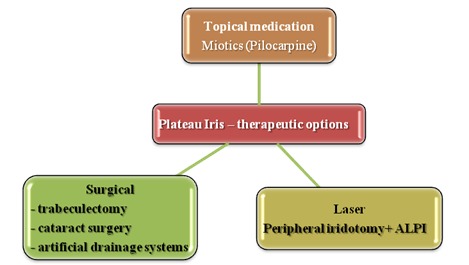
Therapeutic options in plateau iris

Depending of the status of the complications or at the moment of diagnosis, treatment in such patients might differ from case to case. As such, the options are summarized in **Fig. 1**.

The primary treatment modality for patients with Plateau Iris configuration is surgical [**3**]. However, many clinicians will first treat with miotic agents such as pilocarpine to prevent pupillary dilation from leading to surgery. A low dose or dilute pilocarpine can produce the thinning of the iris and facilitate the opening of the angle by pulling the iris away from the trabecular meshwork.

The surgical management is the primary treatment modality in patients with Plateau Iris configuration or syndrome. A patent iridotomy may be therapeutic in reducing risks of angle closure. However, in some patients, laser iridotomy may not significantly alter the anterior chamber depth or anatomy. Even after a successful iridotomy produces what appears to be a well-opened angle, periodic gonioscopy remains crucial because these patients may have incomplete Plateau iris syndrome or the angle may narrow further with age because of the enlargement of the lens.

Argon laser peripheral iridoplasty (ALPI) is the method of choice to effectively open an angle that remains occluded after successful laser iridotomy. It is highly effective, and the effect is maintained for years [**4**]. Yet, even after a successful opening of the angle, regular gonioscopy is mandatory.

## Aim of study

The therapeutic options and functional results in patients with “plateau iris” (syndrome or configuration) in consecutive case series.

## Material and method 

Our study included newly diagnosed patients with acute angle closure by “plateau iris” (configuration or syndrome), between June 2016 and April 2017. All the patients were females, with a mean age of 52.8 +/ - 2.91 years (range between 29–71 years old). The mean decimal visual acuity –BCVA - was 0.81 +/ - 0.19 (range = 0.03–1), 7 were diagnosed with hyperopia and 2 with emmetropia. 

The previous diagnosis in 5 cases was open angle glaucoma/ juvenile glaucoma, 1 patient being misdiagnosed with recurrent uveitis. Documented closed angle glaucoma was met in one patient, whereas in another one symptoms of intermittent angle closure were noticed. 

Baseline mean IOP was 23.8 +/ - 2.45 mmHg, range (12-40 mmHg). Related to topical medication, 39% (7 eyes) had 1 medication, 17% (3 eyes) had 2 topical substances, 11% (2 eyes) were under 3 IOP lowering medications. There was no need for the IOP to be controlled by any topical substance in 6 eyes (33%). 

In 56% of the cases, the diagnosis was Plateau Iris syndrome, in 33% it was Plateau Iris configuration, and 11% were already in the closed angle glaucoma state based on an initial plateau iris configuration.

**Fig. 2:**
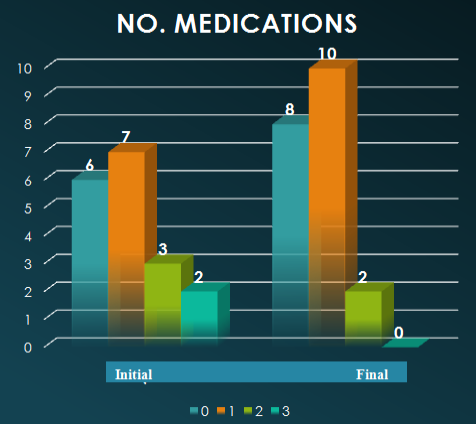
Medication changes after treatment in the current study

**Fig. 3:**
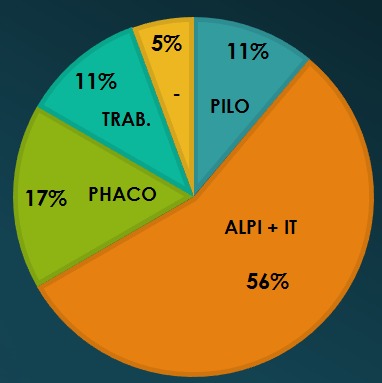
Treatment options in the study group

Papillo-perimetric changes were found in 6 eyes, whereas in 7 cases, disk and structural changes were detected. No changes were detected in 6 eyes. The distribution of cases was as it follows (**Fig. 3**). The final IOP was 15.1 mmHg; in 2 eyes from the same patient, the IOP was not sufficiently controlled and reached levels > 21 mmHg under Pilocarpine (4%), as the patient did not agree with the surgical intervention and preferred this more “non-invasive” type of treatment. After treatment (laser, surgical – trabeculectomy, cataract extraction, miotics administration), the medication changed as shown in **Fig. 2**. The various options in therapy and their distribution in the study group are depicted in **Fig. 3**.

**Clinical case**

For an eloquent clinical example, this study presented the case of a 63-year-old female patient (doctor), complaining about bilateral red, irritated eyes, foreign body sensation, and itching. She had been diagnosed with primary open angle glaucoma for 15 years and had undergone topical IOP lowering treatment with PGA (Travatan®) for a while. Since the inefficacy of this treatment was clear, the treatment regimen was changed to a fixed combination (beta-blocker and carbonic anhydrase inhibitor – Cosopt® in the OD and the same medication plus alpha agonist Brimonidine (Brimonal®) in the OS. 

**Fig. 4:**
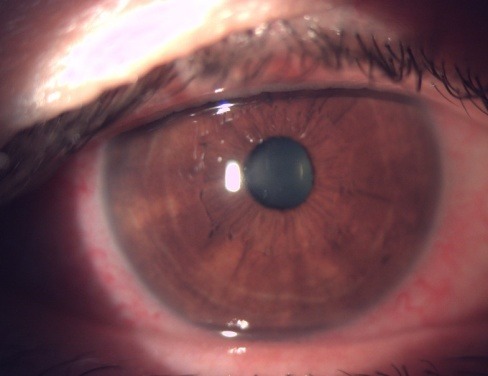
OD - Marked conjunctival congestion

**Fig. 5:**
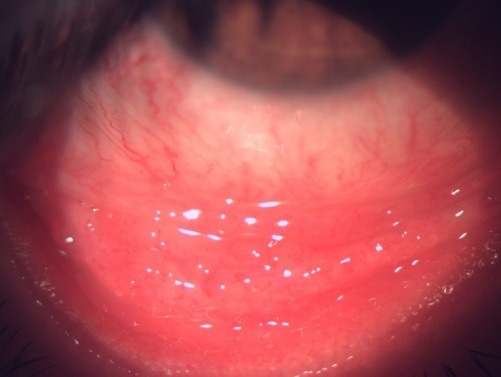
OD - Congestion and inflammatory changes – follicles in the tarsal conjunctiva

**Fig. 6:**
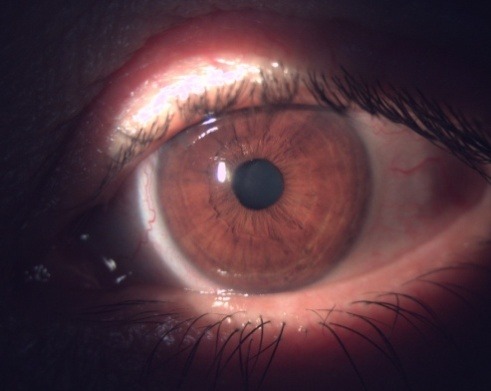
OD - Mild conjunctival congestion

The atypical treatment regimen was emphasized in this patient, as a POAG rarely does not respond to a PGA, and the information regarding the family history was also recollected, as the father of this patient had also been diagnosed with glaucoma at a certain point in his life and followed the treatment. A more thorough anamnesis was restarted and it was found out that the patient had been suffering from Basedow ophthalmopathy and had underwent subtotal thyroidectomy at 30 years old. No exophthalmia was visible, but only an upper lid retraction syndrome. A mild binocular diplopia episode developed when the patient was 48 years old. The substitution hormonal therapy medication was ceased for 2 years. Indeed, the father of this patient had a closed angle glaucoma and he underwent laser treatment in one eye and peripheral iridotomy and trabeculectomy in the other. At the last presentation, the anterior segment showed a conjunctival congestion in both eyes, more visible in the RE, altogether with a marked follicular inflammatory reaction (**Fig. 4**-**6**).

**Fig. 7:**
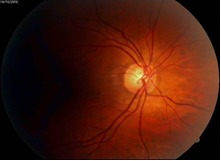
OD Fundus examination. C/ D ratio = 0.5. superior nothing visible

**Fig. 8:**
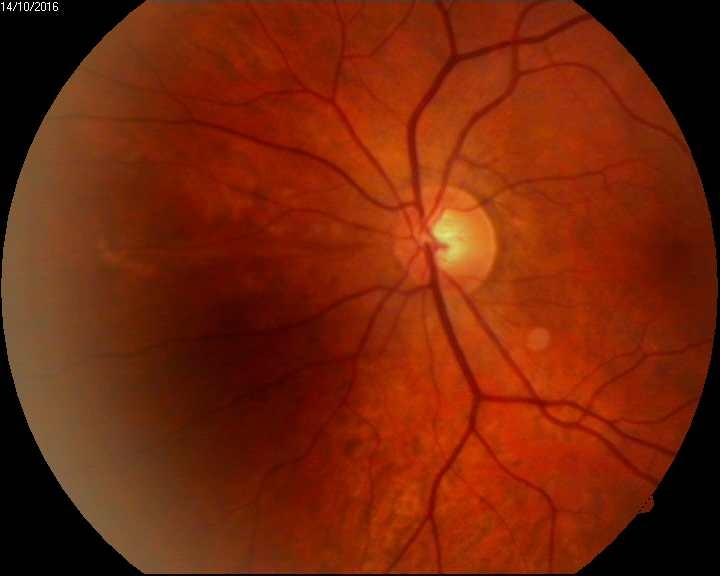
OS Fundus examination. C/ D ratio = 0.4.

IOP in the OD was at that moment 26 mmHg (Cosopt® + Brimonal®) and 19 mmHg in the OS under Cosopt®. Fundus examination can be visualized in **Fig. 7**, **8**.

The visual field examination (HFA II, c24-2, Sita Fast, Zeiss®) showed no functional defect (**Fig. 9a**,**b**) and the OCT (Triton 3000®, Nidek) exam in the OD pointed out the sector RNFL thinning in the superior disk quadrant (**Fig. 10**).

**Fig. 9a:**
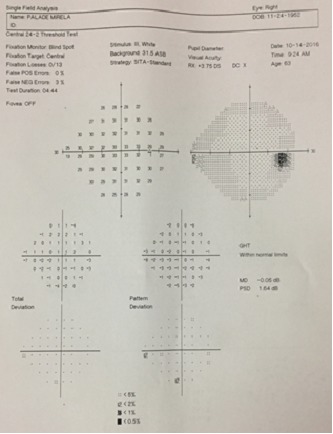
Normal VF in the OD

**Fig. 9b:**
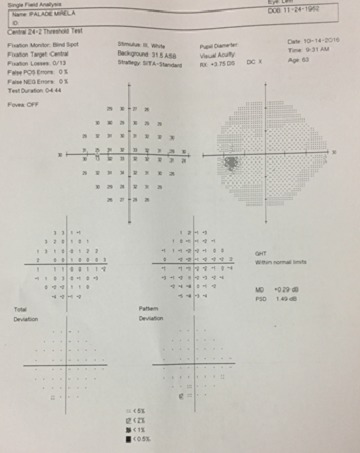
Normal VF in the OS

**Fig. 10:**
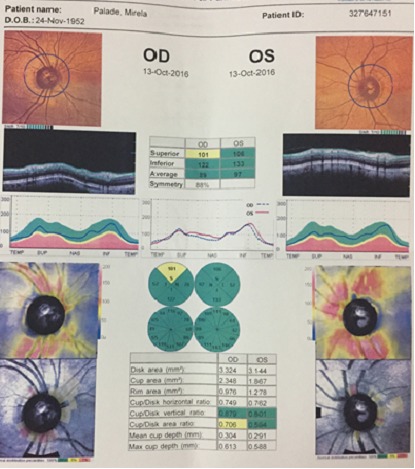
OCT examination

**Fig. 11:**
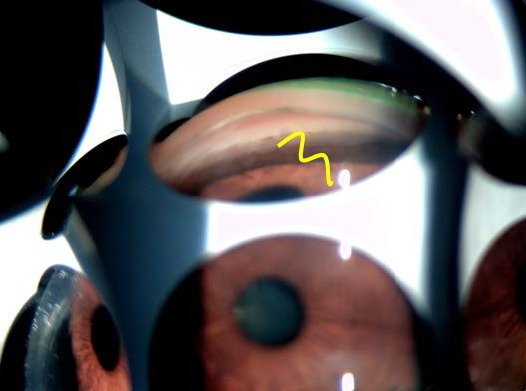
Double hump aspect in indentation gonioscopy in the LE

Gonioscopy revealed an iris with a steep insertion before flattening centrally (iris drapes over the ciliary body producing the double “hump” aspect on indentation) (**Fig. 11**).

In this context, the diagnosis of bilateral “plateau iris” was established and the patient was indicated laser treatment (peripheral argon laser iridoplasty and peripheral iridotomy). After laser treatment was applied, the postoperative evolution was favorable. In the next 7-10 days after Brimonal cessation in the RE, the follicular inflammatory reaction diminished completely. At 6 months, the IOP was 16 mmHg (under topical Cosopt®) and gonioscopy confirmed an open angle bilaterally. This patient was monitored at each 3 months for the IOP, and at each 6 months, she underwent a complete examination, including perimetry, OCT scan, and gonioscopy. As a general advice in her situation, the patient was advised to avoid dim light activities.

## Discussions

Patients with plateau iris tend to be hyperopic, female, and younger than 50 years. In a US-based chart review of the patients under the age of 60, Stieger et al. found the prevalence of plateau iris with recurrent angle-closure symptoms to be 54%, despite the initial iridotomy or iridectomy [5]. In a study from Singapore, Kumar et al. [6] used ultrasound biomicroscopy (UBM) to show that approximately one-third of the patients over the age of 50 with primary angle closure had a plateau iris after laser iridotomy. There is also evidence to suggest that this anatomical predisposition may be familial, with an autosomal dominant inheritance pattern [7]. 

If recognized, the prognosis for patients with plateau iris (syndrome/ configuration) is generally good, if the condition is treated before vision loss occurs. Regular follow up with serial gonioscopy ensures that the proper interventions and treatment modalities are initiated when necessary because angle-closure may develop years after successful iridotomy or iridoplasty. Routine screening for the development of glaucoma should also be performed.

To obtain this, we highly recommend a thorough history of the patient and a high degree of suspicion from the clinician, when conventional medications for glaucoma (PGA in our case) do not work as expected (“plateau iris” configuration/ syndrome vs. true POAG) or when a high discrepancy between structural/ functional damage and the IOP level is met in a glaucoma suspect. Gonioscopy should be mandatory in any glaucoma case we examine and a discrepancy of the AC depth central vs. periphery should alert the clinician regarding certain lines to follow in the diagnosis.

Commonly reported side effects of brimonidine ophthalmic include blurred vision, burning sensation of eyes, drowsiness, eye pruritus, follicular conjunctivitis, headache, local ocular hypersensitivity reaction, ocular hyperemia, stinging of eyes, foreign body sensation, and xerostomia [8]. Therefore, the clinician should be aware of them and act accordingly to the patients’ local tolerance and to the best IOP control.

In conclusion, plateau iris is under diagnosed and it is not that uncommon as previously thought. A careful anamnesis, clinical data and the correct interpretation, gonioscopy and adjunctive imaging technology should be taken into account for each patient with this underlying condition. Based on gonioscopic aspects (closed angle by apposition or by contact, iris insertion and aspect) and the degree of structural and functional deficit, therapeutic options must be individualized for each case. 
